# *Corynebacterium glutamicum* as platform for the production of hydroxybenzoic acids

**DOI:** 10.1186/s12934-018-0923-x

**Published:** 2018-05-12

**Authors:** Nicolai Kallscheuer, Jan Marienhagen

**Affiliations:** 1 Institute of Bio- and Geosciences, IBG-1: Biotechnology, Forschungszentrum Jülich GmbH, 52425 Jülich, Germany; 2 Bioeconomy Science Center (BioSC), Forschungszentrum Jülich GmbH, 52425 Jülich, Germany

**Keywords:** Hydroxybenzoic acids, *Corynebacterium glutamicum*, Shikimate pathway, Metabolic engineering, Protocatechuate, 4-hydroxybenzoate

## Abstract

**Background:**

Hydroxybenzoic acids are industrially relevant aromatic compounds, which also play key roles in the microbial carbon metabolism, e.g., as precursors for the synthesis of cofactors or metal-chelating molecules. Due to its pronounced resistance to aromatics *Corynebacterium glutamicum* represents an interesting platform for production of these compounds. Unfortunately, a complex catabolic network for aromatic molecules prevents application of *C. glutamicum* for microbial production of aromatic compounds other than aromatic amino acids, which cannot be metabolized by this microorganism.

**Results:**

We completed the construction of the platform strain *C. glutamicum* DelAro^5^, in which the deletion of altogether 27 genes in five gene clusters abolished most of the peripheral and central catabolic pathways for aromatic compounds known in this microorganism. The obtained strain was subsequently applied for the production of 2-hydroxybenzoate (salicylate), 3-hydroxybenzoate, 4-hydroxybenzoate and protocatechuate, which all derive from intermediates of the aromatic amino acid-forming shikimate pathway. For an optimal connection of the designed hydroxybenzoate production pathways to the host metabolism, *C. glutamicum* was additionally engineered towards increased supply of the shikimate pathway substrates erythrose-4-phosphate and phosphoenolpyruvate by manipulation of the glucose transport and key enzymatic activities of the central carbon metabolism. With an optimized genetic background the constructed strains produced 0.01 g/L (0.07 mM) 2-hydroxybenzoate, 0.3 g/L (2.2 mM) 3-hydroxybenzoate, 2.0 g/L (13.0 mM) protocatechuate and 3.3 g/L (23.9 mM) 4-hydroxybenzoate in shaking flasks.

**Conclusion:**

By abolishing its natural catabolic network for aromatic compounds, *C. glutamicum* was turned into a versatile microbial platform for aromatics production, which could be exemplarily demonstrated by rapidly engineering this platform organism towards producing four biotechnologically interesting hydroxybenzoates. Production of these compounds was optimized following different metabolic engineering strategies leading to increased precursor availability. The constructed *C. glutamicum* strains are promising hosts for the production of hydroxybenzoates and other aromatic compounds at larger scales.

**Electronic supplementary material:**

The online version of this article (10.1186/s12934-018-0923-x) contains supplementary material, which is available to authorized users.

## Background

Aromatic compounds represent a major class of industrially relevant hydrocarbons, which are for the most part derived from the benzene-, toluene-, ethylbenzene- and xylene- (BTEX) fraction of petroleum [1]. Oxidation of these compounds leads to hydroxybenzoic acids, which serve as building blocks for the production of plastics and fibers, cosmetics, pharmaceuticals, as well as food and feed supplements [2]. However, growing environmental concerns are the main driving force towards establishing sustainable production processes from renewable carbon sources [3]. In this context, microorganisms are recognized as valuable alternative sources for such products as they naturally synthesize a broad range of different aromatic compounds.

The shikimate pathway is primarily responsible for the synthesis of the three aromatic amino acids l-phenylalanine, l-tyrosine and l-tryptophan [4], but intermediates of this pathway are also required for the synthesis of vitamins (e.g., ubiquinone or folate), siderophores or secondary metabolites (e.g., antibiotics) [5–9]. In this context, many shikimate pathway intermediates are first converted to hydroxybenzoates, which then serve as precursor molecules for synthesis of the above-mentioned functional molecules.

Several enzymes catalyzing the initial hydroxybenzoate-forming steps were studied detail, which allows application of these enzymes for the microbial production of various hydroxybenzoates. For the most part, engineering efforts focused on using *Escherichia coli* and *Saccharomyces cerevisiae* as production hosts [10]. The obtained strains were able to produce a broad range of mono- or dihydroxylated benzoic acids, but also phenols (e.g., catechol) or benzaldehydes (e.g., vanillin) became accessible [11, 12]. In other studies, aromatic compounds did not represent the final products but served as precursors for the production of dicarboxylic acids such as *cis*,*cis*-muconic acid and adipic acid with engineered microorganisms [13].

The Gram-positive prokaryote *Corynebacterium glutamicum* has a long standing history in the industrial production of amino acids [14, 15]. In particular l-glutamate and l-lysine are produced at a million ton-scale with this bacterium, but engineered strains for the production of other proteinogenic amino acids are also available [16, 17]. In the course of optimizing aromatic amino acid production in *C. glutamicum*, individual reactions of the shikimate pathway as well as the regulation of this pathway were studied in detail [18]. More recently, *C. glutamicum* was also engineered towards producing shikimate pathway intermediates (e.g., shikimate) and aromatic compounds derived thereof (e.g., *p*-aminobenzoate) [19, 20]. Before that, there was less effort to engineer *C. glutamicum* towards synthesis of such compounds, which can be attributed to the complex network of catabolic pathways for aromatics present in this soil bacterium [21]. *C. glutamicum* is not only capable of degrading aromatic compounds for detoxification purposes, but can also utilize many aromatic compounds such as *p*-coumaric acid, vanillate, *p*-cresol, 3-hydroxybenzoate, 4-hydroxybenzoate and protocatechuate as sole carbon and energy source [22–25]. As *C. glutamicum* was found to have a pronounced resistance to this class of compounds [26], it is reasonable to use *C. glutamicum* as production host. However, any attempt to produce aromatic compounds would require elimination of the catabolic pathways competing for the desired products. In a previous study, we constructed the platform strain *C. glutamicum* DelAro^4^ for the production of plant polyphenols [27]. Key to success was elimination of a catabolic pathway for phenylpropanoids such as cinnamic acid, *p*-coumaric acid and caffeic acid, which represent important precursor molecules for plant polyphenols.

In this study, we abolished additional catabolic pathways in *C. glutamicum* DelAro^4^ and constructed a platform strain, which was subsequently engineered towards producing 2-hydroxybenzoate (2-HB, salicylate), 3-hydroxybenzoate (3-HB), 4-hydroxybenzoate (4-HB) and 3,4-dihydroxybenzoate (protocatechuate, PC). Furthermore, we followed different engineering strategies to ensure increased availability of relevant endogenous precursor molecules for improving overall product synthesis with this organism.

## Methods

### Bacterial strains, plasmids, media and growth conditions

All bacterial strains and plasmids used in this study and their relevant characteristics are listed in Table 1. *C. glutamicum* was routinely cultivated aerobically at 30 °C in brain heart infusion (BHI) medium (Difco Laboratories, Detroit, USA) or defined CGXII medium with 4% glucose as sole carbon and energy source [28]. *E. coli* DH5α used for plasmid constructions was cultivated in LB medium [29] at 37 °C. Where appropriate, kanamycin (50 µg/mL for *E.* *coli* or 25 µg/mL for *C. glutamicum*) or spectinomycin (100 µg/mL for *E. coli* and *C. glutamicum*) was added to the medium. Bacterial growth was followed by measuring the optical density at 600 nm (OD_600_). *C. glutamicum* was grown for 6 - 8 h in test tubes with 5 mL BHI medium on a rotary shaker at 170 rpm (first preculture) and was subsequently inoculated into 50 mL defined CGXII medium with 4% glucose in 500 mL baffled Erlenmeyer flasks (second preculture). The cell suspensions were cultivated overnight on a rotary shaker at 130 rpm. The main culture was inoculated to an OD_600_ of 5.0 in defined CGXII medium with 4% (222 mM) glucose. Heterologous gene expression was induced 1 h after inoculation using the indicated amount of IPTG.

**Table 1:** Strains and plasmids used in this study

Strain or plasmid	Relevant characteristics	Source or reference
*E. coli* strains		
DH5α	F– Φ80*lacZ*ΔM15 Δ(*lacZYA*-*argF*)U169 *recA1 endA1 hsdR17* (rK–, mK +) *phoA supE44* λ– *thi*-*1 gyrA96 relA1*	Invitrogen (Karlsruhe, Germany)
*C. glutamicum* strains		
MB001(DE3)	prophage-free derivate of ATCC 13032 with chromosomal expression of T7 RNA polymerase gene under control of P_*lac*UV5_ (IPTG-inducible)	[65]
DelAro^4^	MB001(DE3) derivative with in-frame deletions of cg0344-cg0347, cg2625-cg2640, cg1226 and cg0502	[27]
DelAro^5^	DelAro^4^ derivative with in-frame deletion of cg3349-cg3354	This study
DelAro^5^ C7	DelAro^5^ derivative with an exchange of the native promoter of the citrate synthase gene *gltA* by the *dapA* promoter variant C7	This study
DelAro^5^ P_O6_-*iolT1*	DelAro^5^ derivative with two point mutations in the promoter of the inositol transporter gene *iolT1,* abolishes repression of *iolT1* by IolR	This study
DelAro^5^ C7 P_O6_-*iolT1*	DelAro^5^ derivative with combined modifications of DelAro^5^ C7 and DelAro^5^ P_O6_-*iolT1*	This study
Plasmids		
pMKEx2	*kan*^r^; *E. coli*-*C. glutamicum* shuttle vector (*lacI*, P_T7_, lacO1, pHM1519 ori_*Cg*_; pACYC177 ori_*Ec*_)	[65]
pMKEx2_aroF*_*qsuB*	pMKEx2 derivate for expression of genes coding for AroF* from *E. coli* (codon-optimized gene) and for QsuB of *C. glutamicum* (native gene)	This study
pMKEx2_aroF*_*irp9*	pMKEx2 derivate for expression of genes coding for AroF* from *E. coli* (codon-optimized) and for Irp9 from *Yersinia enterocolitica* (codon-optimized)	This study
pMKEx2_aroF*_*hyg5*	pMKEx2 derivate for expression of genes coding for AroF* from *E. coli* (codon-optimized) and for Hyg5 from *Streptomyces hygroscopicus* (codon-optimized)	This study
pMKEx2_aroF*_*ubiC*	pMKEx2 derivate for expression of genes coding for AroF* (codon-optimized) and for UbiC (native gene) from *E. coli*	This study
pMKEx2_*aroH*_*ubiC*	pMKEx2 derivate for expression of genes coding for AroH (native gene) and for UbiC (native gene) from *E. coli*	This study
pEKEx3	*spec*^r^; *E. coli*-*C. glutamicum* shuttle vector (*lacI*, P_*tac*_, lacO1, pBL1ori_*Cg*_; pUCori_*Ec*_)	[66]
pEKEx3_*tkt*	pEKEx3 derivate for overexpression of the transketolase gene *tkt* of *C. glutamicum* (the TTG start codon of *tkt* was replaced for ATG)	This study
pK19mobsacB	*kan*^*r*^; vector for allelic exchange in *C. glutamicum* (pK18 oriV_*Ec*_ *sacB* lacZα)	[33]
pK19mobsacB_cg3349-54-updown	pK19mobsacB derivate for the in-frame deletion of cg3349-54	This study
pK19mobsacB_P_O6_-*iolT1*	pK19mobsacB derivate for introducing the mutagenized operator sequence into the *iolT1* promoter region	[48]
pK19mobsacB_ΔP_*gltA*_:P_*dapA*_-C7	pK19mobsacB derivate for exchanging the native *gltA* promoter for the *dapA* promoter variant C7	[50]

*kan*^r^ kanamycin resistance, *spec*^r^ spectinomycin resistance

### Construction of plasmids and strains

Standard protocols of molecular cloning, such as PCR, DNA restriction, and ligation [30] were carried out for recombinant DNA work. Techniques specific for *C. glutamicum*, e.g., electroporation for transformation of strains, were performed as described previously [31]. All enzymes were obtained from ThermoScientific (Schwerte, Germany). Codon-optimized synthetic genes for *C. glutamicum* ATCC 13032 were obtained from LifeTechnologies (Darmstadt, Germany). Genes were amplified by PCR using primers containing unique restriction sites (Table 2). PCR products were then used for cloning of genes into plasmid vectors using the introduced restrictions site. For the assembly of multiple genes and for elimination of undesired internal restriction sites Electra Cloning was used [32]. This cloning strategy is based on the class IIS restriction enzyme *Sap*I, generating 5′-overhangs, which can be freely designed. In-frame gene deletions and nucleotide substitutions in the genome of *C. glutamicum* were performed using the pK19mobsacB system [33] by a two-step homologous recombination method described previously [34]. All constructed plasmids were finally verified by DNA sequencing at Eurofins Genomics (Ebersberg, Germany).

**Table 2:** Oligonucleotides used in this study

Oligonucleotide	Sequence (5′–3′)	Restriction site
del_cg3349-54-up-s	ACCAAGCTTCATTTTCTAGTTGAGATTCATTATCATAACGCTTGACAGTACAACTATGTC	*Hin*dIII
del_cg3349-54-up-as	GCTTCCGGGGTGAATGACGGAGCCGCTGCGGAGGTTCCAGAGCCACCGCGCTG	–
del_cg3349-54-down-s	CGCAGCGGCTCCGTCATTCACCCCGGAAGCTGGCGCTACGCCCTCCTCGAAC	–
del_cg3349-54-down-as	ACCTCTAGATTTCTGTCTTGAGGGTTCGTGGGGGCTG	*Xba*I
check-cg3349-54-s	AAAGGCTCCATGAATTCCTCACGGAGGATCTC	–
check-cg3349-54-as	ACATCACAAGTAGAAACCCGCATTTTCTGTAGTTTTTAC	–
(A)-aroF*-s	TTCGCTCTTCA**AAG**CTGCTTAAGGAGGCTATCTATGACCGATGAACAGGTGCTGATGACCCCAG	*SapI*
(A)-aroF*-as	TACGCTCTTCT**GAT**TTATGCCACGCGTGCGGTCAGCTG	*SapI*
(A)-aroH-s	TTCGCTCTTCA**AAG**TATACCATGGTAAGGAGGTTCAGCATGAACAGAACTGACGAACTCCGTACTGCGCGTATTG	*SapI*
(A)-aroH-as	TACGCTCTTCT**GAT**TTAGAAGCGGGTATCTACCGCAGAGGCGAGTTTTTC	*SapI*
(B)-qsuB-s	TTCGCTCTTCA**ATC**TCTATCAAGGAGGATCGGCATGCGTACATCCATTGCCACTGTTTGTTTGTC	*SapI*
(B)-qsuB-as	TACGCTCTTCT**TCG**TTAGTTTGGGATTCCCCGCTCGAGGTC	*SapI*
(B)-irp9-s	TTCGCTCTTCA**ATC**TCTATCAAGGAGGATCGGCATGAAGATCTCCGAGTTCCTCCACCTGGCAC	*SapI*
(B)-irp9-as	TACGCTCTTCT**TCG**TTACACCATCAGGTATGGTGCAATGGAGGCCAGCTTTTCGCGAGTTTCG	*SapI*
(B)-hyg5-s	TTCGCTCTTCA**ATC**TCTATCAAGGAGGATCGGCATGAACCCATCCTCCTTGGTGCTGAAC	*SapI*
(B)-hyg5-as	TACGCTCTTCT**TCG**TTACATGACCACGCCTTCGATTTCCACGAG	*SapI*
(B)-ubiC-s	TTCGCTCTTCA**ATC**TCTATCAAGGAGGATCGGCATGTCACACCCCGCGTTAACGCAACTG	*SapI*
(B)-ubiC-as	TACGCTCTTCT**TCG**TTAGTACAACGGTGACGCCGGTAAAAACAGTTCTGTTAG	*SapI*
tkt’-s	CATGGATCCAAGGAGGTTCAGCATGACCACCTTGACGCTGTCACCTGAACTTCAG	*Bam*HI
tkt’-as	AGAGCTCTTCA**GCC**CATAGCGTGCTCACGGATACCGAAGTG	*Sap*I
‘tkt-s	TTTGCTCTTCT**GGC**TCCATCCTCAACGGCATTTCCCTCC	*Sap*I
‘tkt-as	TCTGAATTCTTAACCGTTAATGGAGTCCTTGGCCGCTGCCAC	*Eco*RI
pMKEx2_own-s	CCCTCAAGACCCGTTTAGAGGC	–
pMKEx2_own-as	TTAATACGACTCACTATAGGGGAATTGTGAGC	–
pEKEx3-s	GCAAATATTCTGAAATGAGCTGTTGACAATTAATCATC	–
pEKEx3-as	CGTTCTGATTTAATCTGTATCAGGCTGAAAATCTTCTC	–

Restriction sites are underlined; *Sap*I cuts outside of its recognition site, the obtained 5′-overhangs after *Sap*I cleavage used for Electra Cloning are shown in bold. In case of native *tkt* two separate PCR fragments were assembled using Electra Cloning to eliminate an internal *Sap*I restriction site

### LC–MS analysis for quantification of hydroxybenzoates

Hydroxybenzoates 2-HB, 3-HB, 4-HB and PC were quantified in the culture supernatant by LC–MS (Additional file 1: Figures S3–S7) using an Agilent ultra-high-performance LC 1290 Infinity System coupled to a 6130 Quadrupole LC–MS System (Waldbronn, Germany). LC separation was carried out using a Kinetex 1.7u C_18_ 100 Å pore size column (50 mm by 2.1 mm (internal diameter)) (Phenomenex, Torrance, CA, USA) at 50 °C. For elution, 0.1% acetic acid (solvent A) and acetonitrile supplemented with 0.1% acetic acid (solvent B) were applied as the mobile phases at a flow rate of 0.3 mL/min. A gradient was used, where the amount of solvent B was increased stepwise: minute 0–6: 5–30%, minute 6–7: 30–50%, minute 7–8: 50–100% and minute 8–8.5: 100–5%. The mass spectrometer was operated in the negative electrospray ionization (ESI) mode, and data acquisition was performed in selected-ion-monitoring (SIM) mode. Authentic metabolite standards were purchased from Sigma-Aldrich (Schnelldorf, Germany) or from Alfa Aesar (Karlsruhe, Germany). Area values for [M−H]^−^ mass signals were linear up to metabolite concentrations of at least 250 mg/L. Benzoic acid (final concentration 100 mg/L) was used as internal standard. Calibration curves were calculated based on analyte/internal standard ratios for the obtained area values.

## Results

### Pathway design for hydroxybenzoate production

**Fig. 1:**
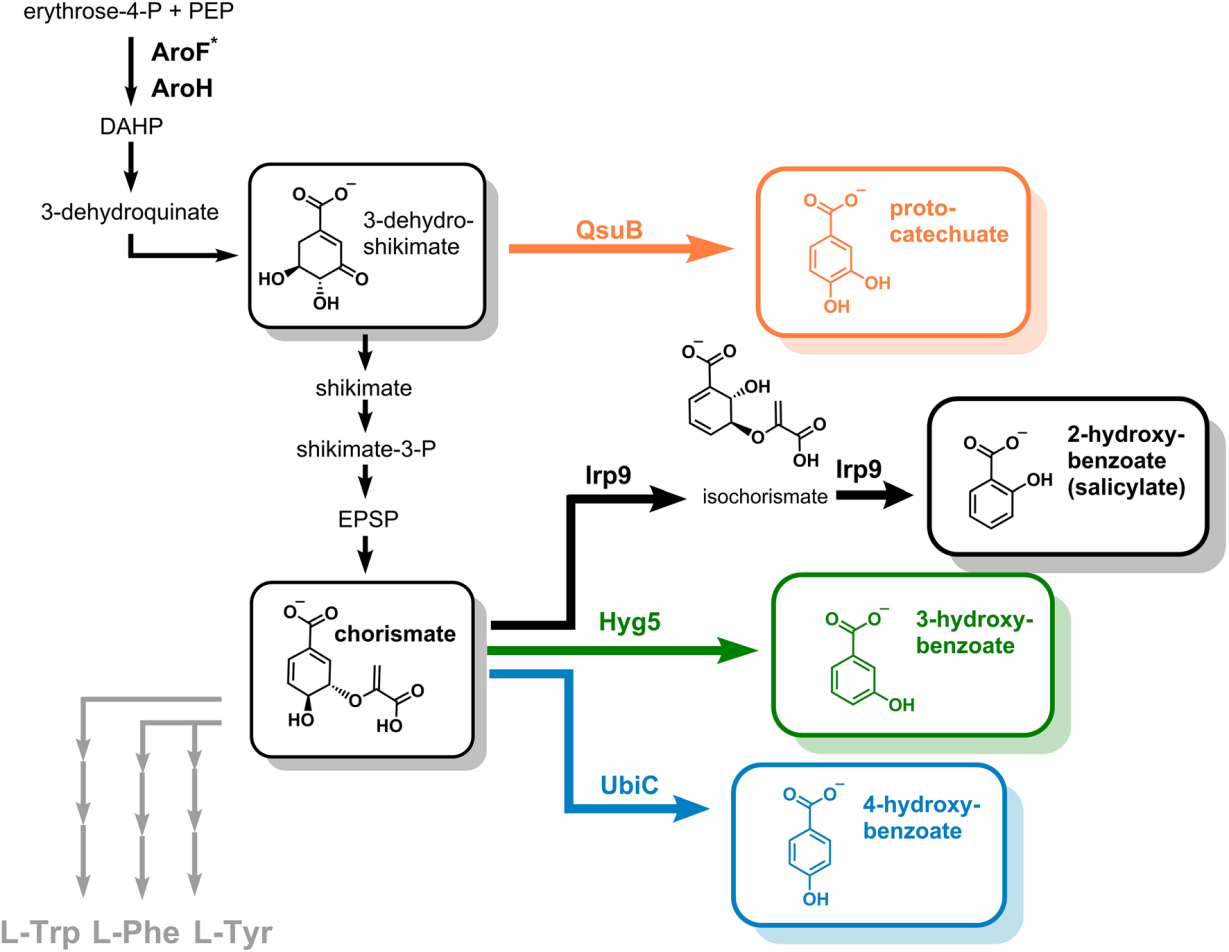
Schematic representation of the shikimate pathway in *C. glutamicum* and additionally introduced enzymatic steps for hydroxybenzoate production. AroF*, AroH: 3-deoxy-d-arabinoheptulosonate-7-phosphate synthase, DAHP: 3-deoxy-d-arabinoheptulosonate-7-phosphate, Hyg5: chorismatase (3-hydroxybenzoate synthase), EPSP: 5-enolpyruvyl-shikimate-3-phosphate, Irp9: isochorismate synthase/isochorismate pyruvate lyase (salicylate synthase), PEP: phosphoenolpyruvate, QsuB: 3-dehydroshikimate dehydratase, UbiC: chorismate pyruvate lyase (4-hydroxybenzoate synthase). Irp9, Hyg5 and UbiC lead to the formation of pyruvate as a second product, which is not depicted

All four desired hydroxybenzoates, PC, 2-HB, 3-HB, and 4-HB can be synthesized from two different intermediates of the shikimate pathway (Fig. 1). PC can be derived from 3-dehydroshikimate by an enzyme-catalyzed dehydration directly yielding this dihydroxybenzoate. *C. glutamicum* possesses the 3-dehydroshikimate dehydratase QsuB (Uniprot entry Q8NT86) normally involved in the pathway for quinate and shikimate degradation, which is known to catalyze this irreversible reaction [35]. The three monohydroxylated benzoates 2-, 3- and 4-HB can be produced from the shikimate pathway intermediate chorismate, but for their synthesis no endogenous enzymes can be recruited (Fig. 1). A detailed analysis of chorismate-converting enzymes identified the bifunctional isochorismate synthase/isochorismate pyruvate lyase (salicylate synthase) Irp9 (Uniprot entry Q9X9I8) in *Yersinia enterocolitica*, which first isomerizes chorismate to isochorismate and subsequently eliminates pyruvate yielding 2-HB [36] (Fig. 1). In *Streptomyces hygroscopicus* the chorismatase (3-hydroxybenzoate synthase) Hyg5 (Uniprot entry O30478) is known to catalyze hydrolysis and concomitant dehydration of the same compound ultimately leading to 3-HB [37]. Finally, 4-HB can be synthesized from chorismate by directly eliminating pyruvate through the enzymatic activity of a chorismate pyruvate lyase (4-hydroxybenzoate synthase) such as UbiC (Uniprot entry P26602), which is involved in ubiquinone biosynthesis in *E. coli* [38].

### An updated *C. glutamicum* strain for the production of aromatic compounds

**Fig. 2:**
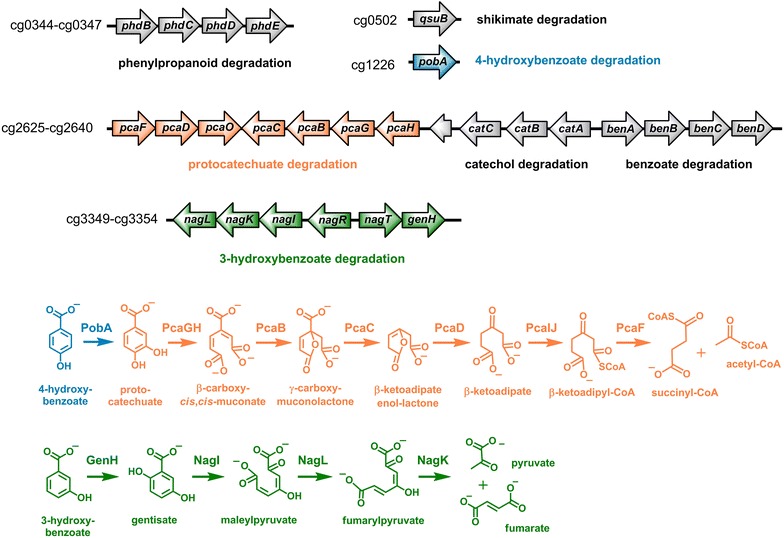
Deleted gene clusters and thus abolished catabolic pathways for aromatic compounds in *C. glutamicum* DelAro^5^. During the construction of *C. glutamicum* DelAro^5^ altogether 27 genes involved in the catabolic network for aromatic compounds and organized in the five clusters were deleted. Among these, genes coding for enzymes involved in the degradation of the depicted hydroxybenzoates are shown in the same color (orange: β-ketoadipate pathway, green: gentisate pathway). No catabolic pathway for 2-hydroxybenzoate is present in *C. glutamicum*. Genes shown in gray code for enzymes involved in catabolic pathways for phenylpropanoids, shikimate, catechol and benzoate

In a preceding study, we observed that activity of the phenylpropanoid degradation pathway (Phd-pathway), a catabolic pathway for phenylpropanoids in *C. glutamicum,* nearly completely abolished production of phenylpropanoid-derived plant polyphenols with this organism [27]. The Phd-pathway ultimately yields different (hydroxy-) benzoic acids, which can be further metabolized by *C. glutamicum* employing the β-ketoadipate pathway. Based on this observation, altogether 21 genes involved in catabolism of aromatic compounds were deleted in the genome of *C. glutamicum* MB001(DE3), a prophage-free derivative of *C. glutamicum* ATCC 13032, including *phdBCDE* (cg0344-47), *qsuB* (cg0502), *pobA* (cg1226) and *pcaFDO*–*pcaCBGH*–cg2633–*catCBA*–*benABCD* (cg2625-40) (Fig. 2). This strain termed *C. glutamicum* DelAro^4^ was successfully applied for plant polyphenol synthesis and represents a suitable starting point for the microbial production of hydroxybenzoic acids as it is unable to degrade 4-HB and PC. However, *C. glutamicum* DelAro^4^ should still be able to metabolize 3-HB by the gentisate pathway, which finally leads to fumarate and pyruvate [39]. The enzymes of the gentisate pathway are encoded by genes of the *nagIKL*–*nagR*–*nagT*–*genH* gene cluster (cg3349-54). In order to prevent any product degradation during the targeted 3-HB production the whole gene cluster was deleted in *C. glutamicum* DelAro^4^ yielding *C. glutamicum* DelAro^5^ (Fig. 2 and Additional file 1: Figure S1). In a cultivation experiment using defined CGXII medium supplemented with 2.8 g/L (20 mM) 3-HB as sole carbon and energy source the reference strain *C. glutamicum* MB001(DE3) reached a final OD_600_ of 10.2 after 24 h (initial OD_600_ was 0.5) and a maximal growth rate of 0.19 h^−1^ (Additional file 1: Figure S2). In the supernatant of *C. glutamicum* MB001(DE3) no 3-HB could be detected at the end of the cultivation. In contrast, *C. glutamicum* DelAro^5^ did not show any growth on 3-HB (Additional file 1: Figure S2) and no decrease of 3-HB concentration in the culture medium could be observed (data not shown).

It has not been tested whether *C. glutamicum* is capable of metabolizing 2-HB. However, cultivation of the reference strain *C. glutamicum* MB001(DE3) in defined CGXII medium supplemented with 2.8 g/L (20 mM) 2-HB showed that *C. glutamicum* is unable to grow with this hydroxybenzoate as sole carbon and energy source. In the culture supernatant obtained after 24 h of cultivation no decrease of the initial concentration of 2-HB could be observed (data not shown).

### Hydroxybenzoate production in *C. glutamicum* DelAro^5^

Establishing hydroxybenzoate production in *C. glutamicum* via the designed pathways required an increased supply of shikimate pathway intermediates by increasing the flux into the shikimate pathway. In *C. glutamicum,* the carbon flux into the shikimate pathway is controlled by allosteric product inhibition of two 3-deoxy-d-arabinoheptulosonate-7-phosphate (DAHP) synthase isoenzymes, which catalyze the initial committed step in this pathway [40]. In order to allow for an increased flux in the pathway the DAHP synthases AroH or AroF from *E. coli* were evaluated for an application during heterologous hydroxybenzoic acid production with *C. glutamicum* (Fig. 1). AroH (Uniprot entry P00887) is only feedback-inhibited by l-tryptophan, but not by the two other aromatic amino acids [41]. In *C. glutamicum*, expression of genes coding for enzymes of the l-tryptophan biosynthetic pathway is repressed by the regulator LtbR when sufficient l-tryptophan is available [42]. Therefore, it was expected that an increased flux into the shikimate pathway does not lead to a significant overproduction of l-tryptophan, which in turn would also inhibit AroH activity. The l-tyrosine-sensitive DAHP synthase AroF (Uniprot entry P00888) from *E. coli* represented a suitable alternative since it could be shown that a truncated version of this enzyme lacking the 11 N-terminal amino acids (designated AroF*) is no longer feedback-regulated by l-tyrosine [43]. For production of different hydroxybenzoates in *C. glutamicum* either *aroH* or aroF* was expressed in combination with a second gene coding for an enzyme capable of converting shikimate pathway intermediates to the desired hydroxybenzoate.

Genes coding for the above-mentioned (heterologous) enzymes QsuB (3-dehydroshikimate dehydratase), Irp9 (salicylate synthase), Hyg5 (3-hydroxybenzoate synthase) and UbiC (4-hydroxybenzoate synthase) were chosen for expression in *C. glutamicum*. In case of aroF*, *irp9* and *hyg5* codon-optimized genes were used. It could be already shown that heterologous expression of the *E. coli* genes *aroH* and *ubiC* yielded functional enzymes in *C. glutamicum* [27, 44]. Thus, both genes were picked for establishing hydroxybenzoate production in *C. glutamicum*. For plasmid constructions, *aroH* or aroF* were cloned in combination with *qsuB*, *irp9*, *hyg5* or *ubiC* as synthetic bicistronic operons into the plasmid pMKEx2 enabling IPTG-inducible expression using the strong T7 promoter. Strength of (heterologous) gene expression was optimized for each of the constructed strains separately. The best-performing combinations of heterologous genes and inducer concentration eventually leading to the highest hydroxybenzoate titers in *C. glutamicum* DelAro^5^ are listed in Table 3.

**Table 3:** Genes and induction conditions leading to the highest obtained hydroxybenzoate product titers in *C. glutamicum* DelAro^5^

Product	DAHP synthase	Codon-optimization	Hydroxybenzoate-forming enzyme	Codon-optimization	IPTG concentration (µM)	Product titer (g/L)
protocatechuate	aroF*	Yes	*qsuB*	No^a^	20	1.47
2-hydroxybenzoate	aroF*	Yes	*irp9*	Yes	20	0.01
3-hydroxybenzoate	aroF*	Yes	*hyg5*	Yes	40	0.26
4-hydroxybenzoate	*aroH*	No	*ubiC*	No	1000	2.32

^a^Endogenous gene (cg0502)

Under optimized conditions, expression of *irp9* in combination with aroF* enabled accumulation of 10 mg/L (0.07 mM) 2-HB in the culture supernatant of *C. glutamicum* DelAro^5^ pMKEx2_aroF*_*irp9* during shaking flask cultivations. In the same strain harboring pMKEx2_aroF*_*hyg5* instead, 0.26 g/L (1.9 mM) 3-HB was produced, whereas heterologous gene expression from the plasmid pMKEx2_aroF*_*qsuB* allowed for a maximal product titer of 1.5 g/L (9.7 mM) PC under the same cultivation conditions. In case of 4-HB, a higher product titer was achieved when, instead of the codon-optimized aroF* gene, the native gene *aroH* was expressed in combination with *ubiC*. In the corresponding strain *C. glutamicum* DelAro^5^ pMKEx2_*aroH*_*ubiC* full induction with 1 mM IPTG allowed for the highest obtained product titer of 2.3 g/L (16.7 mM) 4-HB.

### Engineering of the host towards increased supply of shikimate pathway precursors

In case of all four hydroxybenzoates, expression of two (heterologous) genes was sufficient for enabling product formation with the platform strain *C. glutamicum* DelAro^5^. For PC and 4-HB, g/L-scale product titers could be determined. With the aim to further increase overall product formation, additional engineering of the host metabolism was pursuit to provide increased levels of erythrose-4-phosphate (E4P) and phosphoenolpyruvate (PEP) for the already deregulated initial step of the shikimate pathway. To this end, we tested different strategies for manipulating substrate transport capabilities and key enzymatic activities of the central carbon metabolism, which allow for an increased carbon flux into the shikimate pathway.

**Fig. 3:**
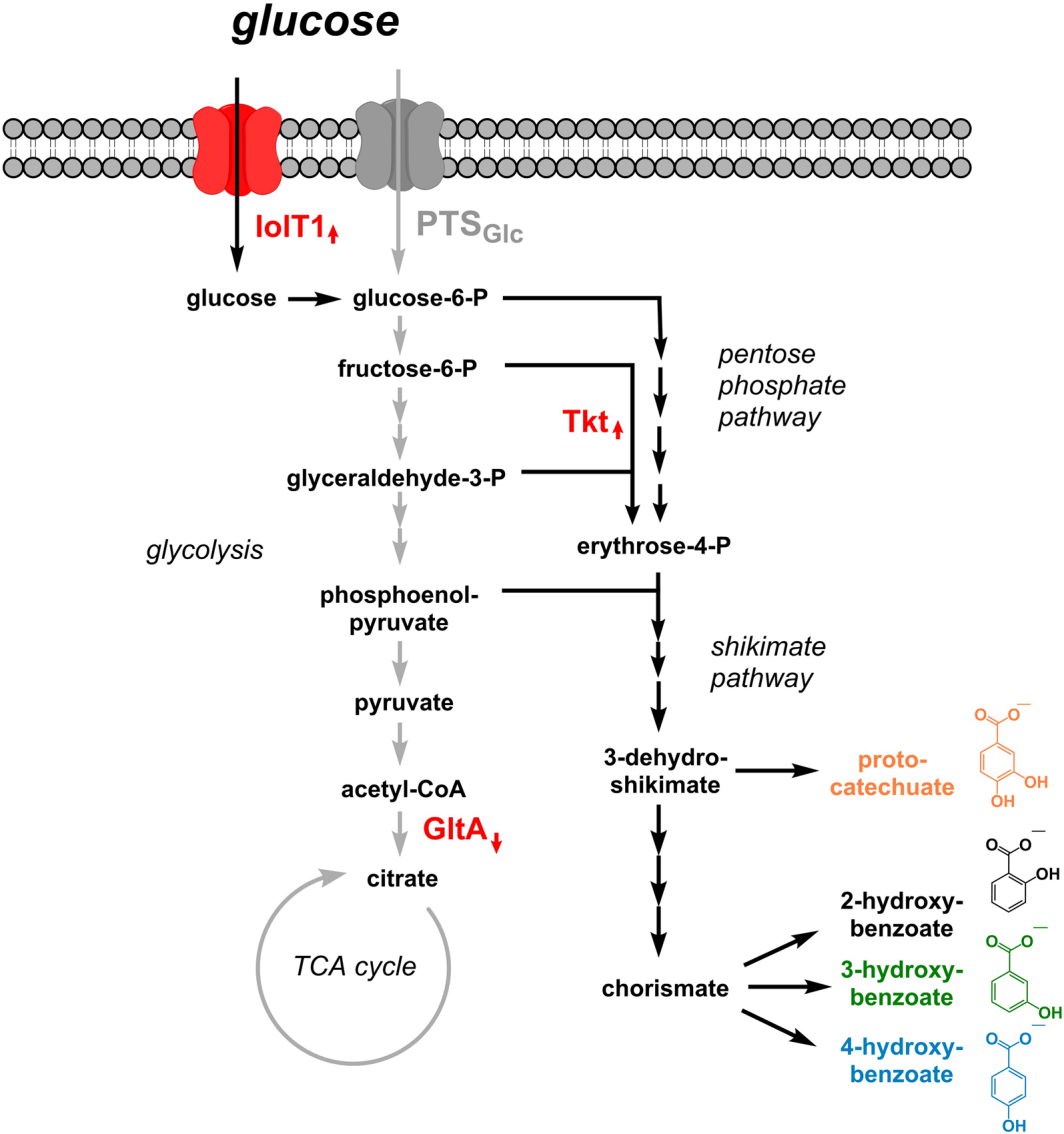
Schematic representation of the main modules of the central metabolism of *C. glutamicum* and introduced modifications for improving hydroxybenzoate synthesis. For an increased production of hydroxybenzoates with *C. glutamicum* DelAro^5^, different genetic modifications were introduced. This included increasing the phosphotransferase-independent sugar import with IolT1 and improving the activity of the transketolase Tkt. In parallel, reducing the GltA-mediated citrate synthase activity reduced the activity of the TCA cycle (see text for details)

In *C. glutamicum*, glucose is taken up by the glucose-dependent phosphotransferase system consuming PEP as phosphate donor [45]. This means that for each glucose molecule taken up and converted to glucose-6-phosphate, one molecule of PEP is simultaneously converted to pyruvate. It was found that the *myo*-inositol/proton symporter IolT1 in *C. glutamicum* is capable of importing glucose by a phosphotransferase-independent transport mechanism [46] (Fig. 3). Expression of the corresponding *iolT1* gene is known to be repressed by the regulator IolR in absence of *myo*-inositol [47]. Recently, IolR-derepression of *iolT1* was realized in *C. glutamicum* by targeted mutagenesis of the IolR-operator sequence (designated O6) in the *iolT1*-promoter region [48]. This genetic modification abolished IolR-binding, allowed constitutive expression of *iolT1* in absence of *myo*-inositol, and allowed for an improved sugar uptake. This modification was also introduced in *C. glutamicum* DelAro^5^ yielding *C. glutamicum* DelAro^5^ P_O6_-*iolT1* with the aim to increase intracellular PEP-availability for product formation.

In a second approach, we aimed at lowering the flux into the tricarboxylic acid (TCA) cycle as this pathway consumes pyruvate and PEP. Pyruvate is converted to the citrate synthase substrate acetyl-CoA by the pyruvate dehydrogenase complex while pyruvate and PEP are also converted to oxaloacetate by anaplerotic reactions. Citrate synthase (CS), which is encoded by the gene *gltA* in *C. glutamicum*, consumes the PEP/pyruvate-derived metabolites oxaloacetate and acetyl-CoA and catalyzes the rate-limiting step for entry of metabolites into the TCA cycle [49] (Fig. 3). It could be already shown that lowering the flux into the TCA cycle by reduced CS activity in *C. glutamicum* leads to an intracellular accumulation of PEP [50]. In order to investigate the effect of a reduced CS activity on hydroxybenzoate production, the native *gltA* promoter was replaced by a variant of the *dapA* promotor (termed C7) controlling expression of the *dapA* gene coding for the dihydrodipicolinate synthase in *C. glutamicum* [51]. Enzyme assays showed that combination of this C7-*dapA* promoter variant with the open reading frame of *gltA* reduced the CS activity by 90% [50]. Thus, we introduced the same modification into *C. glutamicum* DelAro^5^ yielding *C. glutamicum* DelAro^5^ C7.

**Fig. 4:**
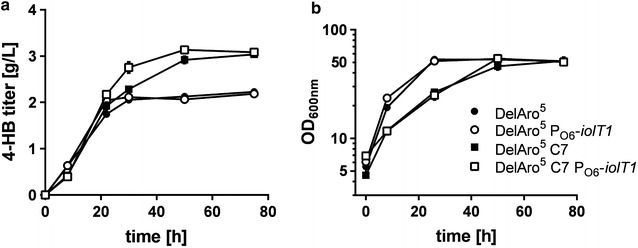
Production of 4-HB with different *C. glutamicum* DelAro^5^ variants. The constructed *C.* *glutamicum* strains harboring pMKEx2_*aroH*_*ubiC* were cultivated in defined CGXII medium with 4% glucose. The heterologous gene expression was induced with 1 mM IPTG one hour after inoculation. 4-HB titers (**a**) and optical densities of the cultures (OD_600_) (**b**) were analyzed at time points indicated. Data represent average values and standard deviation from three biological replicates. In case no error bars are visible, the standard deviation was too small to be displayed

At this stage, we focused on microbial 4-HB production and checked whether the modified strain backgrounds have a positive impact on product formation. Performed cultivation experiments showed that PEP-independent glucose uptake due to deregulated *iolT1*-expression did not increase the overall product titer but the maximum 4-HB concentration of 2.1 g/L (15.2 mM) could be reached eight hours earlier (after 22 h instead of 30 h) (Fig. 4a). The time point at which the maximum 4-HB titer in the shaking flask cultivation could be determined coincided with the depletion of glucose in the medium (data not shown). As expected, *C. glutamicum* DelAro^5^ C7 harboring pMKEx2_*aroH*_*ubiC* showed a 30% lower growth rate as a consequence of the reduced CS activity, but 4-HB production was significantly improved. During the experiments performed, this strain produced 1.9 g/L (13.8 mM) 4-HB within the first 25 h, but production continued until reaching a maximum product titer of 2.9 g/L (21.0 mM) 4-HB after 60 h of cultivation (Fig. 4a).

In the strain *C. glutamicum* DelAro^5^ C7 P_O6_-*iolT1* pMKEx2_*aroH*_*ubiC* both effects, faster product formation and higher product titer, could be successfully combined as this strain was capable of producing 3.1 g/L (22.5 mM) 4-HB within 48 h of cultivation (Fig. 4a). With respect to growth, this new strain behaved like *C. glutamicum* DelAro^5^ C7 pMKEx2_*aroH*_*ubiC* but in principle all engineered strains reached very similar final biomass concentrations as determined by the optical density of the cultures (OD_600_ values ranging from 51 to 55) (Fig. 4b).

In the best-performing strain *C. glutamicum* DelAro^5^ C7 P_O6_-*iolT1* pMKEx2_*aroH*_*ubiC* we additionally tested whether the 4-HB titer can be further increased by enhancing the supply of E4P as second shikimate pathway substrate. The transketolase, a key enzyme of the non-oxidative pentose phosphate pathway, catalyzes the formation of E4P from the glycolysis intermediates fructose-6-phosphate and glyceraldehyde-3-phosphate (Fig. 3) [52]. For additional expression of the native transketolase-encoding gene *tkt*, the plasmid pEKEx3_*tkt* was constructed. *C. glutamicum* DelAro^5^ C7 P_O6_-*iolT1* pMKEx2_*aroH*_*ubiC* pEKEx3_*tkt* was characterized by a maximal growth rate of 0.19 h^−1^ and exhibited a slightly increased 4-HB titer of 3.3 g/L (23.9 mM), which was 10% higher compared to the strain without overexpression of *tkt*.

**Fig. 5:**
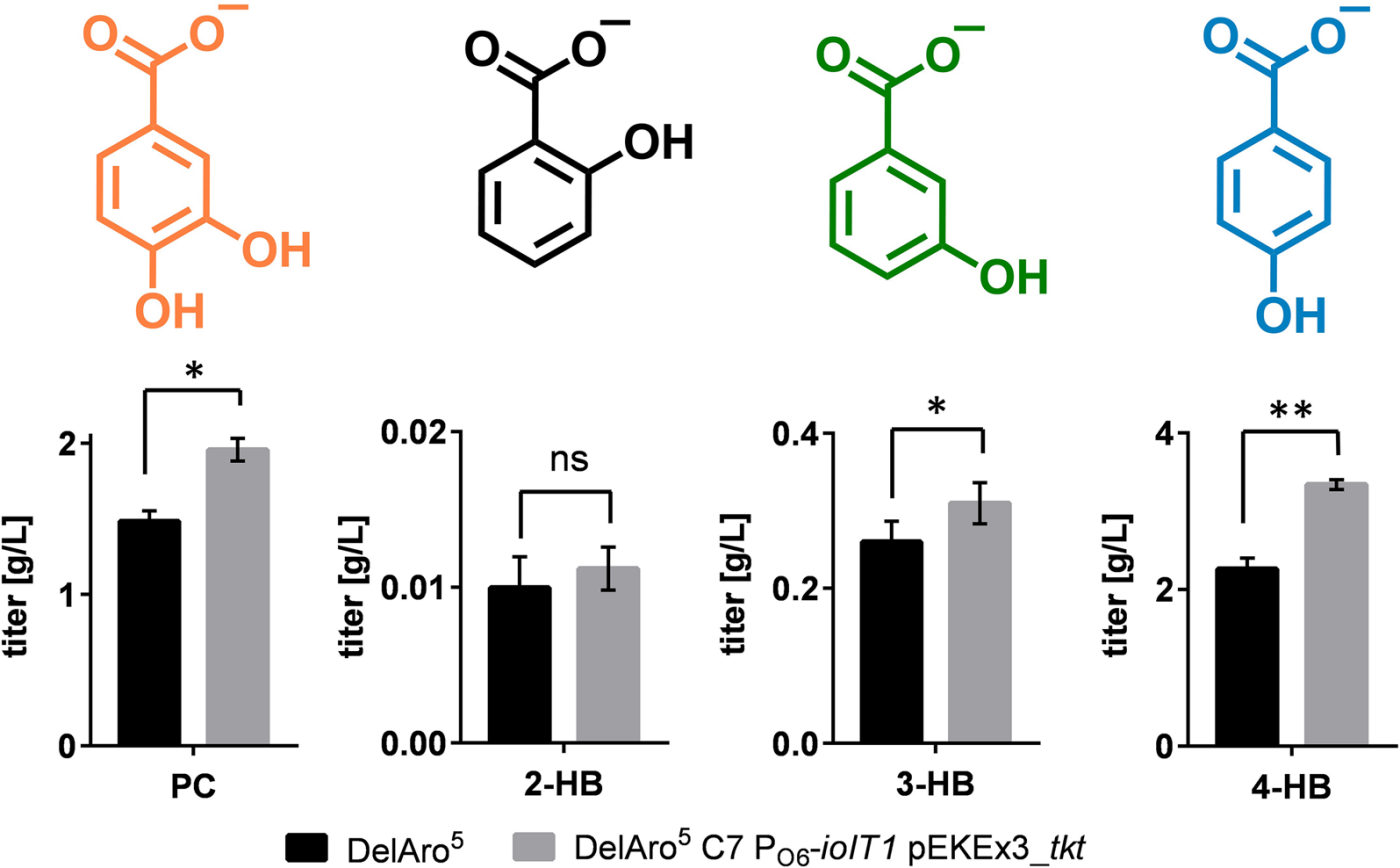
Optimization of hydroxybenzoate production with *C. glutamicum*. The obtained hydroxybenzoate titers obtained with the *C. glutamicum* DelAro^5^ starting strain and *C. glutamicum* DelAro^5^ C7 P_O6_-*iolT1* pEKEx3_*tkt* are shown. Data represent average values of three biological replicates. *p*-values were calculated using student’s *t* test with *p ≤ 0.05 and **p ≤ 0.01 (*ns* not significant)

In order to find out whether this improved strain background also has an impact on PC-, 2-HB-, and 3-HB-production, the respective production plasmids were also introduced into *C. glutamicum* DelAro^5^ C7 P_O6_-*iolT1* pEKEx3_*tkt*. In case of PC and 3-HB, increased product formation could be observed when the respective strains were cultivated in presence of the optimal inducer concentration determined in the initial experiments. The product titers obtained with the optimized strain were 2.0 g/L (13.0 mM) PC and 0.30 g/L (2.2 mM) 3-HB increasing the hydroxybenzoate concentration by 33 and 15%, respectively (Fig. 5). Interestingly, for 2-HB, no significant differences in the obtained product titers could be observed. All strains produced 10 ± 2 mg/L (0.07 ± 0.01 mM) 2-HB independent of the tested strain background (Fig. 5).

Presumably, chorismate was not efficiently converted to the respective hydroxybenzoates in the 2-HB and 3-HB production strains as low concentrations of l-phenylalanine and l-tyrosine (but not l-tryptophan) in the range of 0.08–0.15 g/L (l-Phe: 0.48–0.91 mM, l-Tyr: 0.44–0.82 mM) could be detected (data not shown). Furthermore, LC–MS analysis identified 2-aminobenzoate (anthranilate) as major side product. For instance, 0.6 g/L (4.4 mM) 2-aminobenzoate was found in the best 3-HB-producing strain and 0.8 g/L (5.8 mM) 2-aminobenzoate in the best 2-HB-producing strain. 2-Aminobenzoate is the first intermediate in the l-tryptophan biosynthetic pathway and is directly deduced from chorismate due to the activity of the endogenous anthranilate synthase TrpEG [53].

## Discussion

Although aromatic amino acid-producing *C. glutamicum* strains are available since the 1970s [54, 55], this microorganism was only recently recognized as promising host for the production of other aromatic compounds [19, 27]. Research focused on the identification of catabolic pathways for aromatic compounds in this organism, but catabolic pathways for several aromatic compounds e.g., for 3,5-dihydroxytoluene and naphthalene are still unknown [21–23, 56]. Based on the known catabolic pathways for aromatic compounds, we here constructed *C. glutamicum* DelAro^5^ as a versatile platform organism for the production of aromatic compounds and demonstrated the suitability of this strain by rapidly engineering it towards PC-, 2-HB-, 3-HB- and 4-HB-synthesis. Production of PC in *C. glutamicum* was already reported earlier, but in this case an alternative production route was followed [44]. In this study PC was not obtained directly from 3-dehydroshikimate by its native dehydratase QsuB, but was produced via 4-HB by overexpression of the native 4-hydroxybenzoate 3-hydroxylase gene *pobA*. Bioreactor cultivations of the best-performing strain led to production of 1.1 g/L (7.1 mM) PC from 117 g/L (650 mM) of glucose. The same pathway including *ubiC* and *pobA* was already used earlier in an l-phenylalanine-overproducing *E. coli* strain, in which the competing chorismate mutase/prephenate dehydratase PheA was disrupted. This strategy led to the production of 0.27 g/L (2.0 mM) 4-HB and 0.45 g/L (2.9 mM) PC [57]. Exploitation of the 3-dehydroshikimate dehydratase AroZ in *E. coli* was suitable for PC production via 3-dehydroshikimate with a final product titer of 41 g/L (266 mM) [58]. This titer was achieved in an *E. coli* strain lacking shikimate dehydrogenase activity resulting from a mutation in the encoding gene *aroE*. PC is of particular interest as a precursor for the production of aliphatic polymer building blocks such as the dicarboxylic acids *cis*,*cis*-muconic acid and adipic acid [11]. Currently, there is a great interest in establishing an alternative bio-based platform for dicarboxylic acid production from renewable carbon sources. In this context, the ring-cleaving pathway starting from PC was recognized as a very promising metabolic route for dicarboxylic acid production [13, 59].

*E. coli* was engineered towards producing 2-HB, which also served as precursor for biosynthesis of the polymer building block *cis*,*cis*-muconic acid with this organism [60]. In a more recent study, up to 11.5 g/L (83 mM) 2-HB was produced from glucose using an *E. coli* strain originally engineered for l-phenylalanine production [61]. In this study, production of 2-HB was achieved by the functional introduction of two separate enzymes providing the required isochorismate synthase- and the isochorismate pyruvate lyase-activities instead of using a bifunctional salicylate synthase. In contrast, for the synthesis of 2-HB in *C. glutamicum* in our study, we decided for introducing of such a bifunctional salicylate synthase as this strategy requires the expression of a single heterologous gene instead of two genes. In addition, use of bifunctional enzymes eliminates the need for balancing the expression of both heterologous genes and (potentially) reduces undesired accumulation of isochorismate as pathway intermediate.

However, in the study reporting 2-HB production in *E. coli* mentioned above, production of other aromatic compounds including 3-HB and 4-HB was also achieved [61]. The central carbon metabolism of *E. coli* was engineered towards increased availability of precursor metabolites by combining different modifications in a strain already overproducing l-phenylalanine. Required strain modifications comprised replacement the PEP-dependent phosphotransferase sugar uptake system by a PEP-independent permease and simultaneous inactivation of *pykF* and *pykA* (pyruvate kinase) as well as of *pheA* (chorismate mutase/prephenate dehydratase) and *tyrA* (chorismate mutase/prephenate dehydratase), which are both involved in the conversion of chorismate to l-phenylalanine and l-tyrosine, respectively. Heterologous expression of the required genes for hydroxybenzoate synthesis in the resulting strain allowed for product titers of 2.2 g/L (15.9 mM) 3-HB and 1.8 g/L (13.0 mM) 4-HB.

In a very recent study, 4-HB production with *C. glutamicum* was achieved by overexpressing seven genes coding for enzymes of the shikimate pathway [62]. The obtained strain was additionally engineered by deleting genes encoding for the pyruvate kinase, the dihydroxyacetone phosphate phosphatase, the lactate dehydrogenase and the 3-dehydroshikimate dehydratase to avoid accumulation of pyruvate, dihydroxyacetone, lactate and PC as side products. Functional introduction of a highly 4-HB-resistant 4-HB synthase from *Providencia rustigianii* into this strain allowed for the accumulation of 36.6 g/L (265 mM) 4-HB during aerobic growth-arrested bioreactor cultivations. Any degradation of 4-HB was prevented by deletion of the 4-hydroxybenzoate 3-hydroxylase gene *pobA*. The obtained 4-HB titer with the constructed *C. glutamicum* strain is more than tenfold higher than the maximal titer of 3.3 g/L (23.9 mM) obtained in this study, but when considering the very different cultivation and production conditions, obtained product titers cannot be compared. The maximum titer of 36.6 g/L was determined during growth-arrested bioreactor cultivations of pre-grown cells in complex medium in the presence of 7.2% glucose. In contrast, the product titer of 3.3 g/L 4-HB was obtained in shaking flask cultivations with defined medium lacking any complex media ingredients and using only 4% glucose as sole carbon and energy source. However, when only comparing the strain performance of both studies during shaking flask cultivation similar titers of 20-25 mM (2.7 - 3.5 g/L) 4-HB were achieved upon expression of *ubiC* from *E. coli*.

The product 4-HB is not only an industrially relevant precursor for parabens, which find an application as preservatives in cosmetics and pharmaceuticals [63], but can be also serve as a precursor molecule for the production of plant-derived phenylpropanoids and polyphenols with *C. glutamicum* using a novel synthetic pathway not known to nature [64]. Combination of the heterologous 4-HB-forming pathway with the synthetic pathway for phenylpropanoid and polyphenol production would require selection of *C. glutamicum* DelAro^5^ as host strain as the catabolic pathways for 4-HB and the phenylpropanoid intermediates are abolished in this strain. This underlines that *C. glutamicum* DelAro^5^ developed in this study represents a promising strain for the production of many different natural and non-natural aromatic compounds.

For enabling a large-scale production of aromatic compounds with *C. glutamicum*, additional metabolic engineering work is required. This work should primarily focus on balancing the host metabolism and the heterologous pathways, e.g., for preventing side product formation. In particular during 2-HB- and 3-HB-synthesis, activity of the respective heterologous enzymes converting chorismate appeared to be rate-limiting, which presumably promoted the observed side product formation. Furthermore, identification and engineering of rate-limiting enzyme-catalyzed reaction steps either fueling or as part of the shikimate pathway will contribute to increase overall product titers.

## Additional file

**Additional file 1: Figure S1.** Confirmation of the successful deletion of the nag-gene cluster by PCR. **Figure S2.** Cultivation of *C. glutamicum* strains with 3-hydroxy benzoate as sole carbon and energy source. **Figure S3.** LC-MS analysis of microbially produced PC. **Figure S4.** LC-MS analysis of microbially produced 3-HB. **Figure S5.** LC-MS analysis of microbially produced 4-HB. **Figure S6.** LC-MS analysis of microbially produced 2-HB. **Figure S7.** Extracted chromatograms for m/z of benzoate and of 2-HB.

## Abbreviations

2-HB: 2-Hydroxybenzoate (salicylate)
3-HB: 3-Hydroxybenzoate
4-HB: 4-Hydroxybenzoate
BTEX: Benzene-, toluene-, ethylbenzene- and xylene-fraction of petroleum
CS: Citrate synthase
E4P: Erythrose-4-phosphate
PC: 3,4-Dihydroxy-benzoate (protocatechuate)
PEP: Phosphoenolpyruvate
TCA: Tricarboxylic acid cycle
