# Supplementary material for: Corynebacterium glutamicum as platform for the production of hydroxybenzoic acids
Source: Microb Cell Fact. 2018 May 12;17:70. doi: 10.1186/s12934-018-0923-x (PMC5948850; doi:10.1186/s12934-018-0923-x)
Supplement: Supplementary file 1 — Additional file 1: Figure S1. Confirmation of the successful deletion of the nag-gene cluster by PCR. Figure S2. Cultivation of C. glutamicum strains with 3-hydroxy benzoate as sole carbon and energy source. Figure S3. LC-MS analysis of microbially produced PC. Figure S4. LC-MS analysis of microbially produced 3-HB. Figure S5. LC-MS analysis of microbially produced 4-HB. Figure S6. LC-MS analysis of microbially produced 2-HB. Figure S7. Extracted chromatograms for m/z of benzoate and of 2-HB. [file 12934_2018_923_MOESM1_ESM.pdf]

Additional File 1

Corynebacterium glutamicum as platform for the production of hydroxybenzoic acids

Nicolai Kallscheuer[1,2] and Jan Marienhagen[1,2*]

[1]Institute of Bio- and Geosciences, IBG-1: Biotechnology, Forschungszentrum Jülich GmbH, Jülich D-52425, Germany

[2]Bioeconomy Science Center (BioSC), Forschungszentrum Jülich GmbH, Jülich D-52425, Germany

[*]Corresponding author
Dr. Jan Marienhagen, phone +49 2461 61 2843, e-mail j.marienhagen@fz-juelich.de

This additional file contains:

Fig. S1	Confirmation of the successful deletion of the nag-gene cluster by PCR
Fig. S2	Cultivation of *C. glutamicum* strains with 3-hydroxy benzoate as sole carbon and energy source
Fig. S3	LC-MS analysis of microbially produced PC
Fig. S4	LC-MS analysis of microbially produced 3-HB
Fig. S5	LC-MS analysis of microbially produced 4-HB
Fig. S6	LC-MS analysis of microbially produced 2-HB
Fig. S7	Extracted chromatograms for m/z of benzoate and of 2-HB

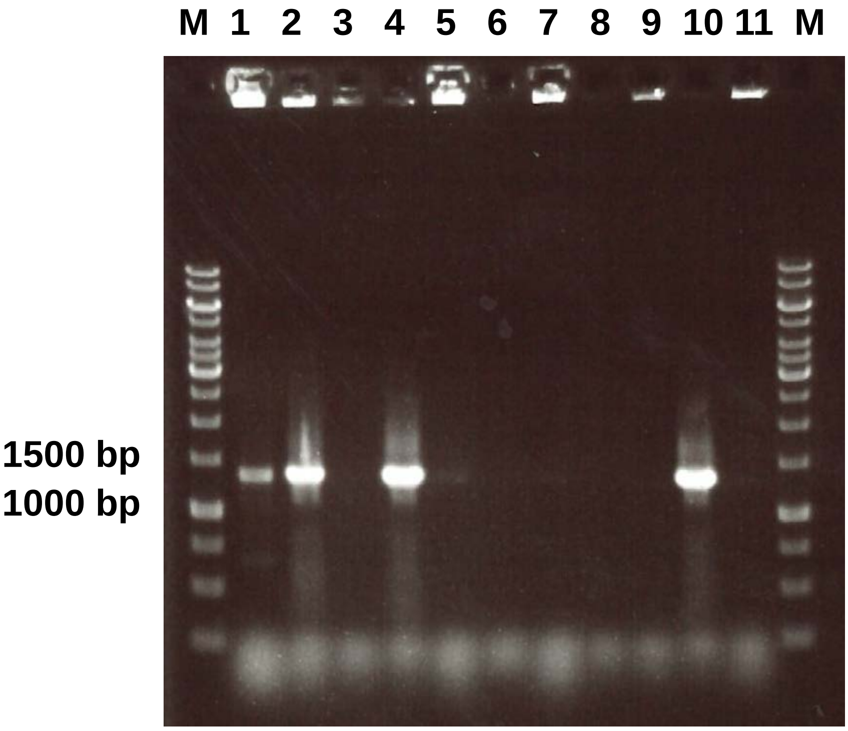

Additional File 1: Fig. S1. Confirmation of the successful deletion of the *nag*-gene cluster by PCR

The deletion of cg3349-cg3354 (*nagIKL-nagR-nagT-genH*) in *C. glutamicum* DelAro[4] yielding *C. glutamicum* DelAro[5] was confirmed by colony-PCR and subsequent agarose gel electrophoresis. For this purpose, oligonucleotides *check_cg3349-54-s* and *check_cg3349-54-as* were designed bind up- and downstream of the flanking regions targeted during for homologous recombination, respectively. Successful deletion of the five genes corresponded to a PCR product of 1332 bp, whereas no PCR product was expected for the wild-type situation as the resulting PCR product of 7701 bp was too long to be amplified. Clone 4 (lane 4) was selected for subsequent strain engineering towards microbial production of aromatic compounds.

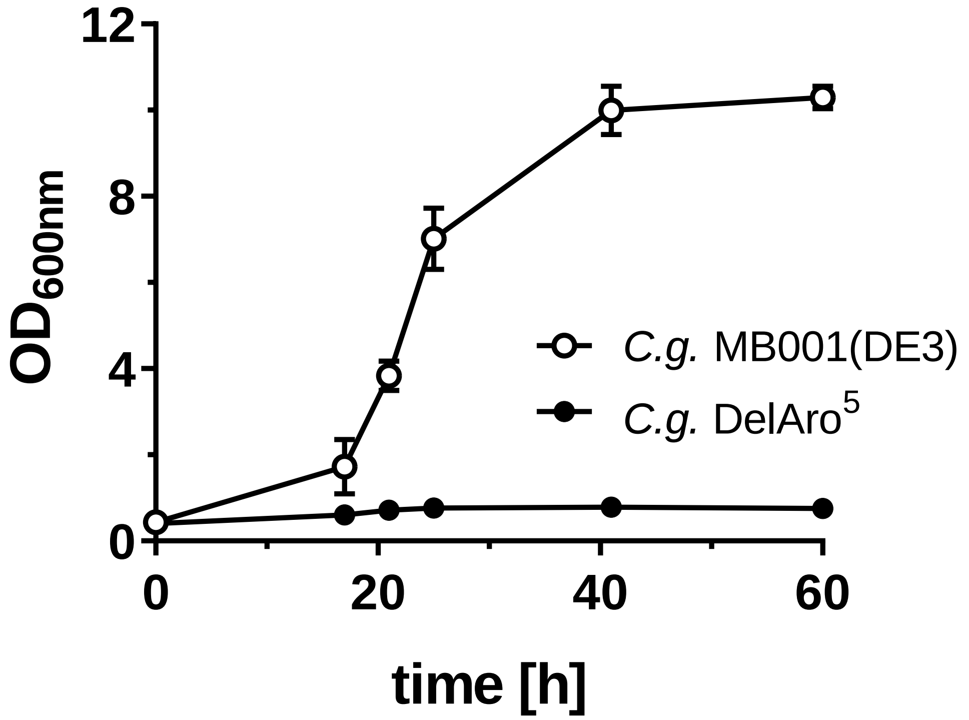

Additional File 1: Fig. S2. Cultivation of C. glutamicum strains with 3-hydroxy-benzoate as sole carbon and energy source

Successful deletion of cg3349-3354 was also confirmed by cultivation of *C. glutamicum* DelAro^5 and of the reference strain *C. glutamicum* MB001(DE3) in defined CGXII medium with 20 mM 3-hydroxy benzoate as sole carbon and energy source.

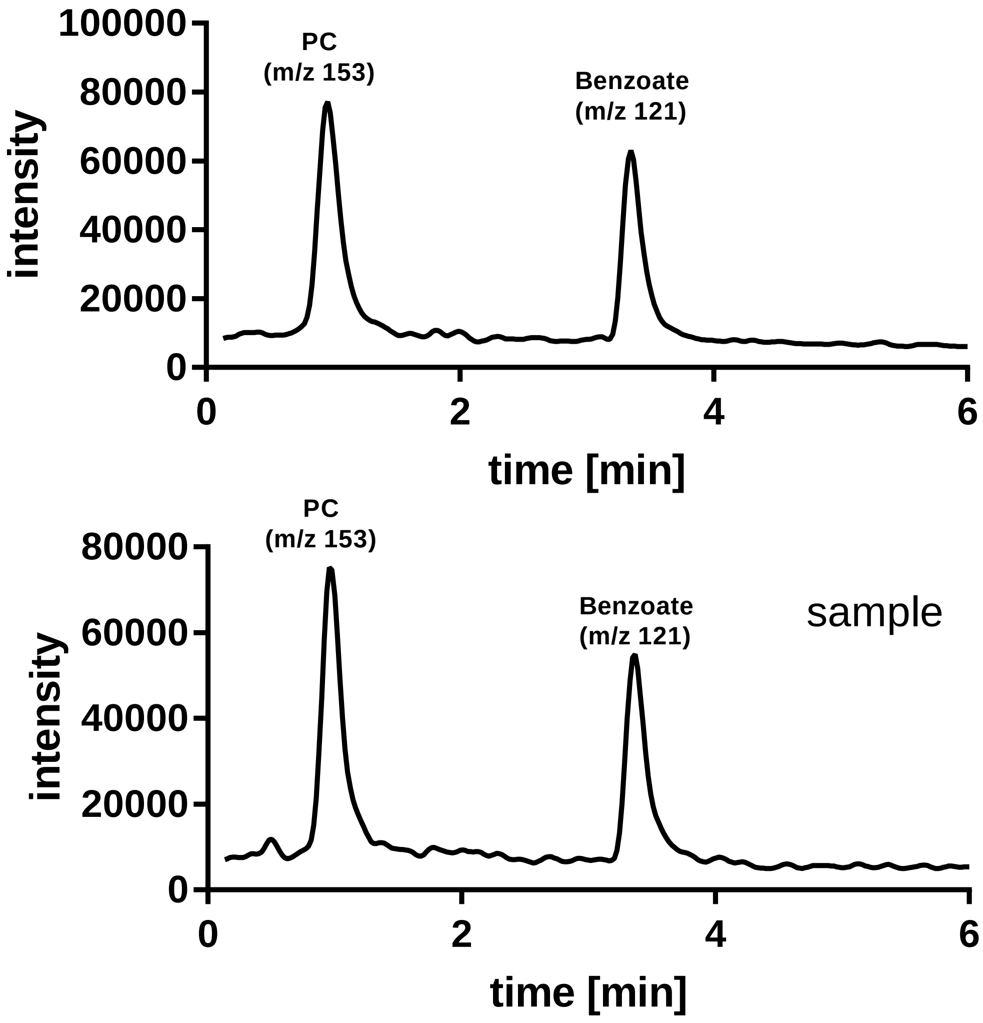

Additional File 1: Fig. S3. LC-MS analysis of microbially produced PC

The concentration of PC (m/z = 153, negative mode) produced by the constructed *C. glutamicum* strains was analyzed by LC-MS analysis in the culture supernatant. The retention times of the identified peaks were identical to the retention times of commercially available standards of the respective compound (upper chromatogram). Benzoate (m/z 121) was used as an internal standard.

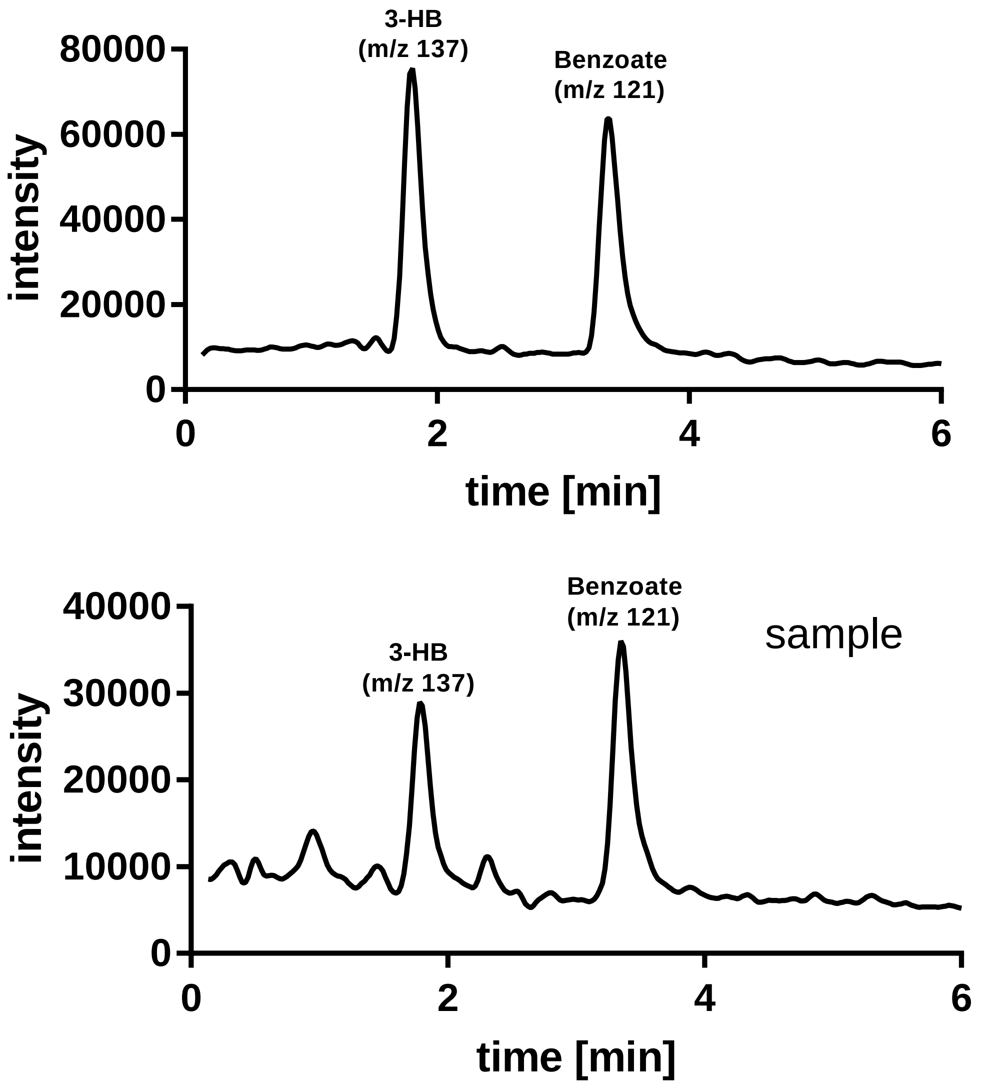

Additional File 1: Fig. S4. LC-MS analysis of microbially produced 3-HB

The concentration of 3-HB (m/z = 137, negative mode) produced by the constructed *C. glutamicum* strains was analyzed by LC-MS analysis in the culture supernatant. The retention times of the identified peaks were identical to the retention times of commercially available standards of the respective compound (upper chromatogram). Benzoate (m/z 121) was used as an internal standard.

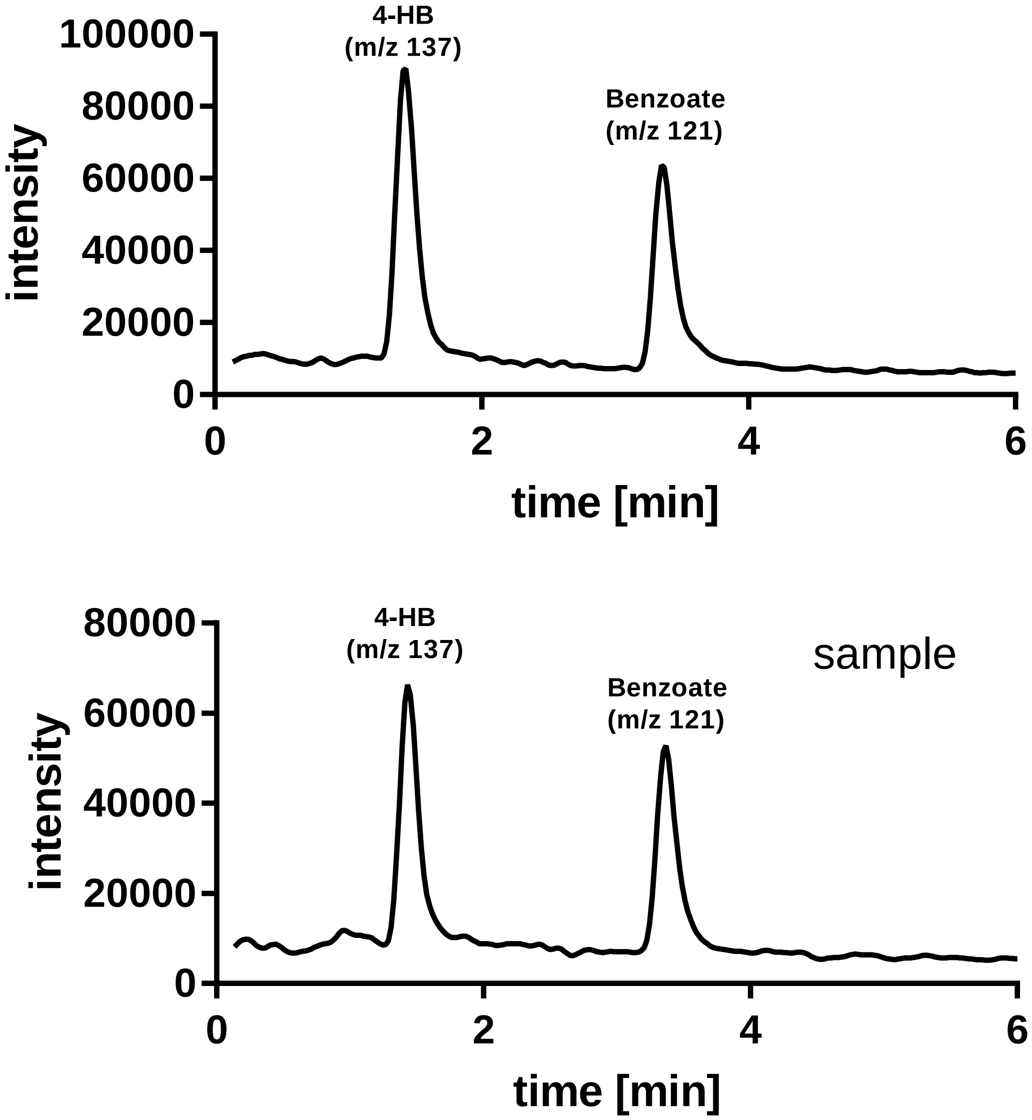

Additional File 1: Fig. S5. LC-MS analysis of microbially produced 4-HB

The concentration of 4-HB (m/z = 137, negative mode) produced by the constructed *C. glutamicum* strains was analyzed by LC-MS analysis in the culture supernatant. The retention times of the identified peaks were identical to the retention times of commercially available standards of the respective compound (upper chromatogram). Benzoate (m/z 121) was used as an internal standard.

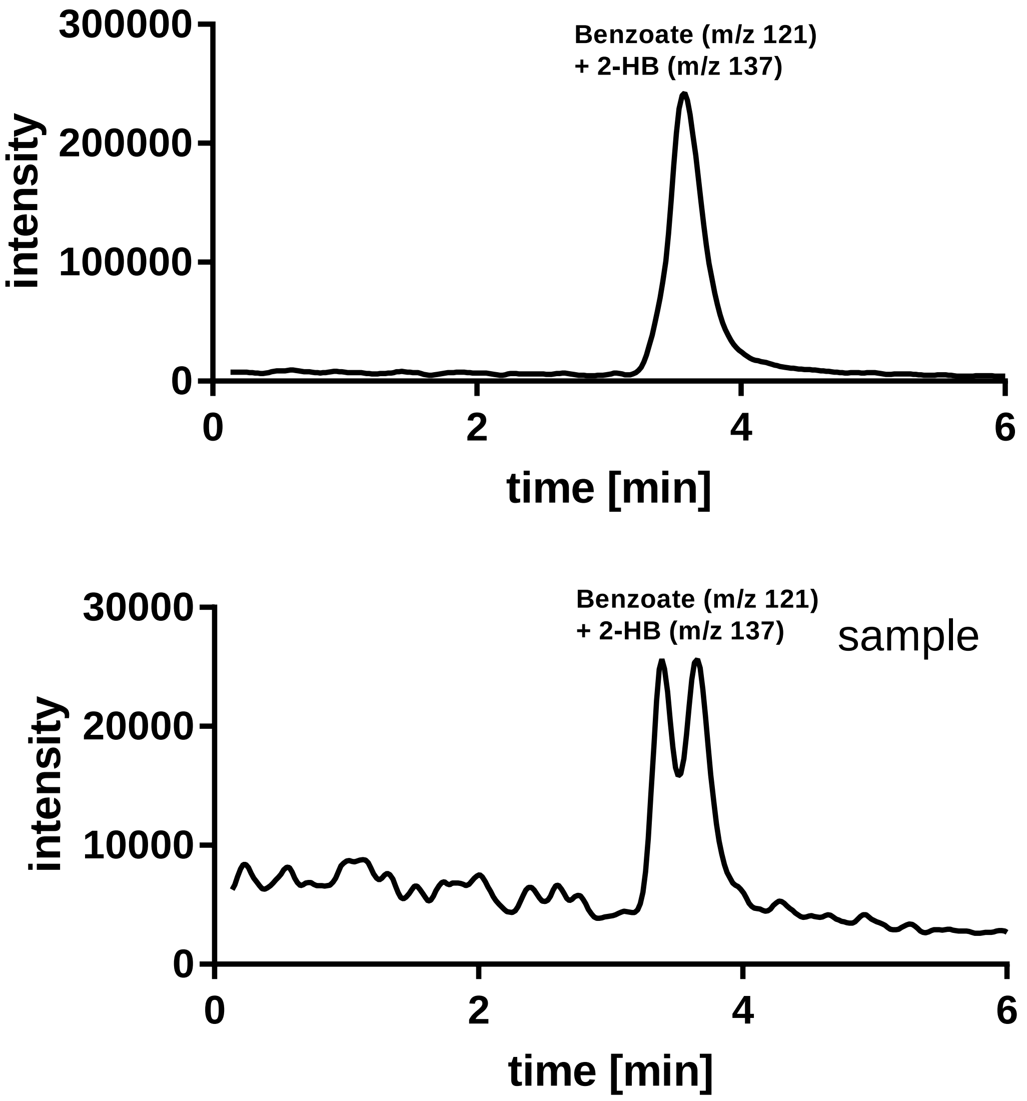

Additional File 1: Fig. S6. LC-MS analysis of microbially produced 2-HB

The concentration of 2-HB (m/z = 137, negative mode) produced by the constructed *C. glutamicum* strains was analyzed by LC-MS analysis in the culture supernatant. The retention times of the identified peaks were identical to the retention times of commercially available standards of the respective compound (upper chromatogram). Benzoate (m/z 121) was used as an internal standard. The retention times of benzoate and 2-HB were very similar so that only one peak was observed during analysis of the metabolite standard. For an unambiguous identification of the respective peaks and accurate calculation of the peak areas extracted chromatograms for m/z of 2-HB and of benzoate are shown for standard and sample in Fig. S7.

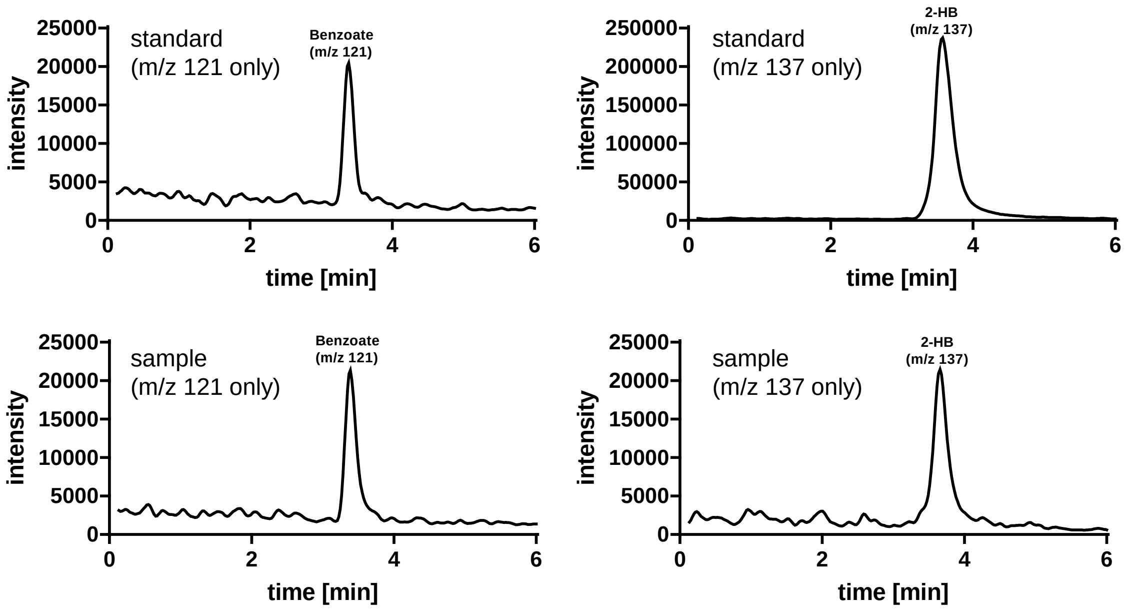

Additional File 1: Fig. S7. Extracted chromatograms for m/z of benzoate and of 2-HB

The similar retention times of 2-HB (3.65 min) and of the internal standard benzoate (3.38 min) did not allow an accurate calculation of the peak area. Therefore, the accurate peak areas for these two compounds were obtained from extracted chromatograms for m/z 121 (benzoate) and m/z 137 (2-HB). The merged chromatograms of m/z 121 and 137 yield the obtained chromatogram shown in Fig. S6.
